# Association between Changes in the Patterns of Antinuclear Autoantibodies during Immune Checkpoint Inhibition Therapy and the Development of Severe Immune Related Adverse Events

**DOI:** 10.3390/ijms232012641

**Published:** 2022-10-20

**Authors:** Leticia Alserawan, Geòrgia Anguera, Carlos Zamora Atenza, Jorgina Serra López, Laura Martínez-Martínez, Mariona Riudavets Melià, Ivana Sullivan, Andrés Barba Joaquin, Margarita Majem Tarruella, Silvia Vidal

**Affiliations:** 1 Immunology-Inflammatory Diseases, Biomedical Research Institut Sant Pau, IIB Sant Pau, 08026 Barcelona, Spain; 2 Department of Medical Oncology, Hospital de la Santa Creu i Sant Pau, 08026 Barcelona, Spain; 3 Department of Immunology, Hospital de la Santa Creu i Sant Pau, Biomedical Research Institute Sant Pau (IIB Sant Pau), 08026 Barcelona, Spain

**Keywords:** immune-related adverse events (irAEs), anti-PD-(L)1 blocking agents, antinuclear autoantibodies (ANA), predictive biomarkers, immune checkpoint inhibitors

## Abstract

Immune-related adverse events (irAEs) are unpredictable autoimmune-like toxicities induced by immune checkpoint inhibitors (ICI). irAEs are a consequence of a breakdown in self-tolerance. ICIs can induce autoantibody formation, and the presence of antinuclear autoantibodies (ANAs) has been reported in patients who developed irAEs. Our goal was to compare ANA patterns by indirect immunofluorescence at different timepoints before (baseline) and after the initiation of ICI treatment and to analyze the role of ANA pattern changes as predictors of irAEs. This is a 2-year-follow-up prospective study of 152 consecutive patients with solid tumors treated with anti-PD-(L)1 blockade agents. They were included from September 2018 until March 2020 in the Hospital de la Santa Creu I Sant Pau (Barcelona, Spain). We grouped patients into three groups: ANA de novo (patients who showed new ANA patterns at any time after ICI initiation), ANA (ANA positive at baseline without changes in the ANA patterns after initiation of treatment) and non-ANA (ANA negative at baseline and after ICI initiation). We did not find any association between the appearance of ANAs and irAE rates or the number and types of irAEs. However, patients in the ANA de novo group showed higher severe irAE rates (grade ≥ 3) than the other groups. Additionally, in most of the patients with severe irAEs (83.3%), changes in ANA patterns preceded irAE onset. In conclusion, we found ANA induction during ICI therapies in 22 patients and our results suggest that the appearance of ANAs may predict the severity of the irAE.

## 1. Background

Immune checkpoints, such as PD-1 (Programmed cell death 1 receptor) and CTLA-4 (Cytotoxic T lymphocyte antigen 4), are the main regulatory molecules of the immune system that maintain immune homeostasis and self-tolerance. PD-1 is a T cell surface receptor that recognizes and binds to the endogenous ligands PD-L1 and PD-L2 on antigen-presenting cells (APCs) and on tumor cells. Its recognition by ligands attenuates T lymphocyte effector function, including proliferation and cytokine production. Similarly, CTLA-4 is a co-inhibitory molecule that blocks T-cell activation by direct competition with CD80/CD86 on APCs. Blocking these receptors restores T effector functions and enhances endogenous anti-tumor immunity [1,2].

Immune checkpoint inhibitors (ICI) improve the survival of patients with solid tumors, such as melanoma and lung cancer [3,4,5,6]. Despite their efficacy, these treatments can induce novel toxicities in the form of autoinflammation or autoimmunity, which are commonly named immune-related adverse events (irAEs) [2,7]. irAEs are diverse and unpredictable, potentially affecting almost every organ system at any moment. They can occur in up to 80% of patients treated with ICI [8,9]. While some patients may experience a single irAE, others may develop several autoimmune toxicities, either simultaneously or separated temporally [8]. Up to 25% of irAEs can be severe and may require immunosuppressors or treatment disruption [9,10]. The identification of patients at risk of developing irAEs, especially the most severe forms, is crucial for prompt management in order to prevent complications or prevent treatment suspension. For this reason and due to their unpredictability, there is an urgent need to find appropriate meaningful biomarkers predictive of irAEs.

Emerging studies indicate that irAEs are a consequence of a breakdown in self-tolerance mediated at least in part by antigen-specific T-cell responses, B cells, autoantibodies and cytokines [7,8,10]. This breakdown in central and/or peripheral tolerance might result in the maturation of autoreactive T-cells and B-cells and their subsequent differentiation into antibody-secreting plasma cells [11]. An increase in the levels of plasma cells and autoantibodies during ICI treatment has been reported [12,13].

Autoantibodies are involved in the pathogenesis and inflammation of some traditional autoimmune diseases [14]. The presence of autoantibodies does not necessarily imply the development of an autoimmune disease but could be considered a biological marker for autoimmune predisposition [15,16]. There is a similarity between autoimmunity and irAEs in terms of their clinical and biological phenotypes, and this is consistent with the risk of flares of pre-existent autoimmunity in patients who receive ICI agents [17,18,19,20]. Therefore, as in several autoimmune disorders, autoantibody screening could be useful in the prediction, diagnosis and/or prognosis of irAEs.

Antinuclear antibodies (ANAs) comprise a spectrum of autoantibodies directed against the nuclear and cytoplasmic components of normal human cells. ANAs are laboratory markers that support the diagnosis of autoimmune-mediated diseases such as systemic lupus erythematosus, systemic sclerosis, Sjögren disease and autoimmune myositis [21,22]. They are not specific markers since positive ANA tests have also been obtained for chronic infectious diseases, cancer and after medications [23]. Additionally, ANAs can also be present in healthy individuals, though usually at low titers (less than 5% of the healthy population at serum dilution 1:160) [24]. Nevertheless, ANAs are valuable diagnostic criteria when accompanied by other clinical manifestations.

The presence of ANAs in patients who receive ICI therapies and develop irAEs has been reported. Most published studies have focused on analyzing the predictor role of pre-existing ANAs prior to ICI therapy and their association with the risk of irAEs [25,26,27]. Seroconversion to ANA positive after ICI initiation has been also reported [28,29]. However, the association between ANA positivity and irAEs is still controversial. Our purpose was to compare ANA patterns at different time points during ICI treatment in patients diagnosed with advanced solid tumors and to associate the changes in patterns with irAE features. First, we analyzed the differences in ANAs between pre- and serial post-treatment, and then we studied the association between ANA changes and the development and rates, type, number and severity of the irAEs.

## 2. Results

### 2.1. Patients’ Characteristics

Ninety-five (70.9%) patients were male and 39 (29.1%) were female. The mean age was 67.7 (±1.01) years. The most frequent tumor type was non-small cell lung cancer (NSCLC) in 91 (67.9%) patients, followed by 20 (14.9%) cases of melanoma, 10 (7.5%) of renal cancer, nine (6.7%) of head and neck cancer, and four (2.9%) of bladder cancer. In regard to the stage, 82 cases of NSCLC (90.1%), 16 of melanoma (80%), 9 of renal cancer (90%) and 100% of head and neck and bladder cancer cases were metastatic (stage IV). Nine cases of NSCLC (10.1%) and 1 of renal cancer (10%) were stage III locally advanced and 4 cases of melanoma (20%) were stage III resected. None of the patients included in the study had a previous history of autoimmune disease.

Sixty-four (47.7%) patients received anti-PD-(L)1 blockade agents as a first-line treatment and 57 (42.5%) as a second-line treatment or beyond. Five (3.7%) patients received ICI as an adjuvant therapy and eight (5.9%) patients received it as manteinance therapy after chemoradiation. Anti-PD-(L)1 blockade agents were administered as monotherapy in 104 patients (77.6%), in combination with another immunotherapy agent in 19 (14.2%) patients and with chemotherapy in 11 (8.2%) patients.

Based on ANA presence, patients were divided into three study groups: the first group (ANA de novo) included patients with or without ANA in baseline samples who developed new ANA patterns after ICI initiation (*n* = 22); the second group (ANA) included patients with ANA in all serial samples without changes in patterns (*n* = 37); and the third group (non-ANA) included patients without ANA in all serial samples (*n* = 75). The baseline characteristics of patients segregated into groups are detailed in Table 1. There were no significant differences in sex, age, tumor type and treatment between the three groups, except for atezolizumab. A higher frequency of ANA de novo patients was found in the subgroup of patients with a high expression of PD-L1.

### 2.2. IrAE Characteristics

Eighty-one patients (60.4%) developed irAEs: the median number of iRAEs was two (IQR 1–2.5). 39 (29.1%) patients developed 1 irAE and 42 (31.3%) patients developed two or more irAEs. The maximum number of irAEs developed per patient was seven. A total of 154 irAEs developed in 81 patients. Mild irAEs (grades ≤ 2) were more frequent than severe irAEs (grades ≥ 3): according to CTCAE, 85 (55.2%) irAEs were grade 1; 47 (30.5%) were grade 2; 15 (9.7%) were grade 3 and 7 (4.5%) were grade 4. No grade 5 irAEs were developed.

With regard to the type of irAE, dermatological toxicities including rash and pruritus were the most frequent manifestations and were observed in 56 (41.8%) of patients, followed by endocrine manifestations in 21 (15.7%) patients, hepatitis in 12 (8.9%) patients and colitis in 10 (7.5%) patients.

**Figure 1:**
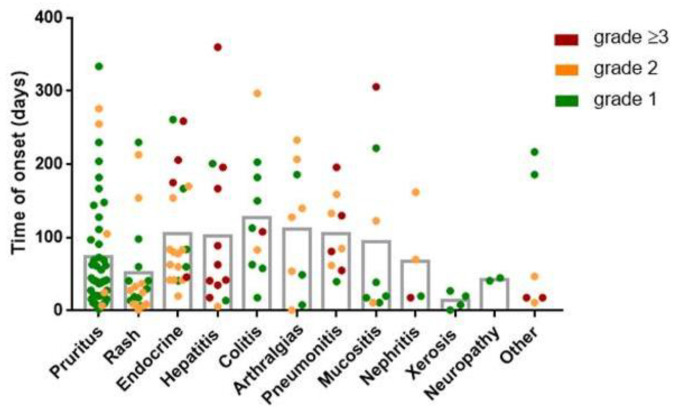
Description of immune-related adverse events. The time of onset and severity of the irAEs occurred in all patients in the study, according to the type of irAE. Each point represents an irAE (*n* = 154). Types of irAE are ordered from the highest to the lowest frequency. Endocrine irAEs included hypothyroidism (*n* = 12), hyperthyroidism (*n* = 4, hypophysitis (*n* = 4), and diabetes mellitus (*n* = 1). Other irAEs include grade ≥ 3 myocarditis (*n* = 1) and thrombocytopenia (*n* = 1), grade 2 vasculitis (*n* = 1) and psoriasis (*n* = 1), and grade 1 myalgia (*n* = 1) and vitiligo (*n* = 1).

The earliest toxicity was rash; the median time of onset was 28 (IQR 9–50.5) days. In contrast, the latest toxicities developed were artralgias and colitis, which appeared after 128 (28.5–196) and 110.5 (61.75–187.3) days. The characteristics of the irAEs are shown in Figure 1. The most severe irAEs (grade ≥ 3) affect hepatic, respiratory and endocrine systems.

### 2.3. ANA Pattern Appearance during ICI Therapy

**Figure 2:**
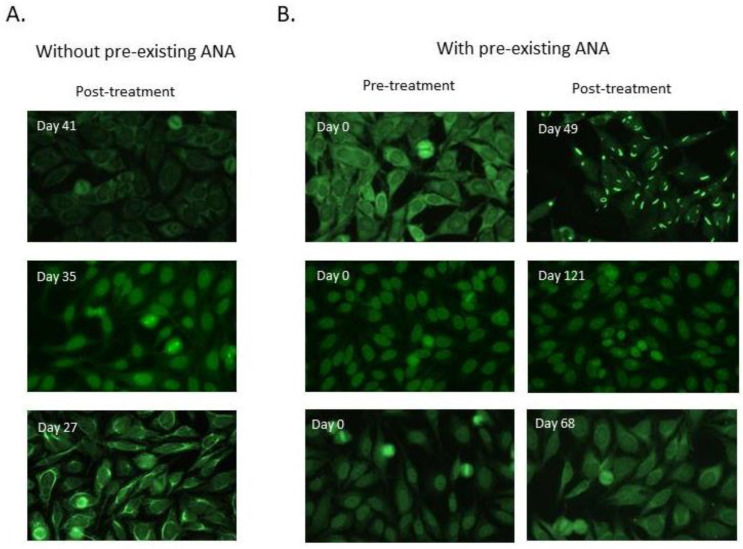
Indirect immunofluorescence images that show differences in ANA patterns in pre- and post-treatment serum samples of patients who developed ANA during ICI treatment. Representative IIF images of pre-treatment and post-treatment serum samples from patients who developed ANA patterns de novo after ICB initiation (**A**) without pre-existing ANA and (**B**) with pre-existing ANA. Days passed after ICI initiation in post-treatment samples are indicated in each image. Negative pre-treatment samples are not represented.

Forty-five (33.6%) patients had ANA at baseline samples, and 59 (44%) patients had ANA after ICI initiation. The comparison of IIF patterns between samples collected at baseline and after ICI initiation revealed that 22 (16.4%) patients (14 patients who were ANA negative and eight patients who were ANA positive in baseline samples) developed ANAs de novo after ICI initiation. Representative IIF images are shown in Figure 2.

The individual characteristics of patients from the ANA de novo group are detailed in Table 2. The most frequent ANA de novo pattern was the nuclear speckled pattern in 10 patients, followed by the cytoplasmic fibrillar in five patients. Other less frequent ANA patterns were: nucleolar (three patients), cytoplasmic speckled pattern (three patients), intercellular bridge pattern (two patients), rods & rings (one patient), NuMa-like pattern (one patient), spindle fibers (one patient) and cytoplasmic dots (one patient). ANA de novo appeared at different times after ICI initiation; the median time of appearance was 51 (IQR 40.5–111.5) days. As the table shows, 63.6% of the patients in this group developed irAEs, with pruritus as the most frequent irAE (six patients), followed by hepatitis (five patients). The ANA patterns developed were not specific to irAE types. No common clinical features (solid tumor type, ICI agent, type, severity or number of irAE manifestations) were found among patients who developed the same ANA pattern, nor in patients who developed ANA de novo.

### 2.4. Association between ANA Development and IrAEs

**Figure 3:**
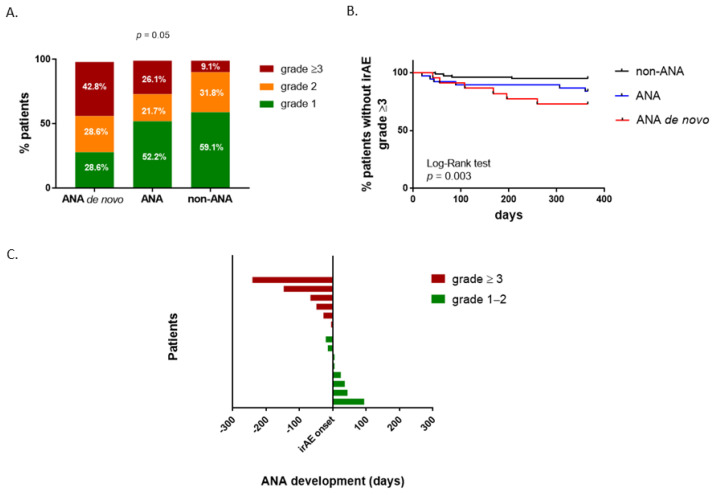
Comparison of irAE onset and severity between groups. (**A**) Association of severity of irAEs and ANA development. (**B**) Staircase graphs show the percentage of patients without grade ≥3 irAE over time in patients grouped according to ANA presence during ICI treatment. (**C**) Relationship between the moment of appearance of new ANAs during ICB treatment and the moment of irAE onset. Each bar represents one patient. Log-rang Mantel–Cox test was used for analysis of patient groups during the 365 days of follow-up.

Preexisting ANA at baseline was not associated with irAE development. There were 53 patients without preexisting ANA who developed irAEs and 17 patients with preexisting ANA who did not develop irAEs during the follow-up period (*p* = 0.85). Considering only patients who developed irAEs, 28 (34.5%) patients presented with ANA at baseline and 37 (45.6%) had ANA at any time point. There was no difference in irAE prevalence between the groups of patients: ANA de novo, ANA and non-ANA groups (rates 63.6%, 62.2% and 58.7% respectively). Among the groups, there were no differences in either the number of irAEs developed or the time of appearance and the type of irAE (Supplementary Figure S1). However, the ANA de novo group showed higher rates of severe irAE than the other two groups (*p* = 0.05) (Figure 3A).

When we compared the presence of irAEs during the 12 months of follow-up among the three groups, we found differences in the time of grade ≥3 irAEs onset (Figure 3B,C). We did not observe differences in the time of irAE onset between patients with and without preexisting ANA, regardless of the grade of irAEs (data not shown). Additionally, in the ANA de novo group, we found that in the 83.3% patients who suffered grade ≥3 irAEs (five patients), the irAE manifestation was preceded by ANA appearance (Figure 3C). The other 16.6% of the patients (one patient) developed ANA at the same time as irAE. In contrast, in patients with grade 1–2 irAEs (eight patients), ANA development was produced after irAE manifestation (six patients, 75%). The mean days of ANA appearance were 88.6 (±36.6) days prior to grade ≥3 irAEs and 20.3 (±13.4) days after grade 1–2 irAEs.

In addition, we compared the treatment responses in the three groups. ANA de novo group showed superior OS and significant superior PFS (*p* = 0.01). These results were also confirmed in the NSCLC population (the major and most homogeneous population in our cohort) (Supplementary Figure S2).

## 3. Discussion

To the best of our knowledge, this is the first study that specifically associates ANA pattern development after ICI initiation with the clinical features of irAEs according to three study groups: ANA de novo, ANA and non-ANA. Our results showed the development of ANAs in a subset of patients after ICI initiation. Although these patients did not share common clinical characteristics and we did not find an association between the presence of ANAs and the development of irAEs in patients who received ICI, the ANA de novo group showed higher severe IRAE rates than the other two groups. In most patients with severe irAEs (83.3%), ANA development preceded irAE onset, which suggests that ANA screening could help predict the severity of the irAE.

We showed that 16.6% of patients receiving ICI developed ANAs de novo, suggesting, in line with other authors, that checkpoint blockade is related to the T-cell-dependent activation of autoreactive B cells and autoantibody production [30,31]. It has been postulated that cancer cells can induce an immunological response, resulting in the production of tumor-associated autoantibodies [32,33,34]. However, the possibility that antibody production is a spontaneous consequence of tumoral antigenicity, independent of ICI treatment, cannot be completely ruled out.

We did not find differences in irAE prevalence between patients with and without pre-existing ANAs nor between patients with and without ANAs in serial samples after ICI initiation. These results are consistent with other studies [25,26,27,29]. Considering the clinical similarity between autoimmune diseases and irAEs, the lack of association between the presence of ANA and the risk of irAE could be explained by two main reasons. First, the presence of ANA could be considered a biological marker of autoimmune predisposition, but it does not necessarily entail the development of clinical manifestations. Second, as in certain autoimmune diseases, some irAEs may be not ANA-related but rather due to cell-mediated immune mechanisms or autoantibody-mediated mechanisms different from ANA [35]. In line with this second reason, other authors have correlated the presence of autoantibodies with higher irAE rates [36,37], though these studies considered a broader spectrum of different autoantibodies, not specifically ANAs, which may explain the different results.

ANA development was not associated with higher rates of irAEs, the number of irAEs or the type of irAE. However, a trend toward its association with the severity of irAE was found. In addition, the ANA de novo group showed a higher OS and PFS. Considering that the development of irAEs is associated with immune checkpoint blockade response [38], this is not unexpected since checkpoint blockade can enhance anti-tumoral T cell response at different grades and severe irAEs could reflect stronger immunological T cell responses. In contrast with our results, another study did not find any association between autoantibody induction and a higher grade of irAEs [27]. This discordance may be due to the heterogeneity in the antibodies tested and the patient cohorts. Other authors associated seroconversion to positive ANAs, extractable nuclear autoantibodies (ENA) or anti-smooth muscle autoantibodies (ASMA) in the first 30 days after ICI initiation with better treatment outcomes and a higher risk of irAEs [28]. Similar to our results, other studies did not find a significant association between ANA development and irAE rates [27,30,39].

We showed that ANA appearance preceded irAE manifestations in 83.3% of the patients who suffered grade ≥ 3 irAEs. This finding suggests that the early appearance of ANA could be a useful predictive biomarker of severe irAEs. In concordance with our results, Das et al. showed that patients with early B-cell changes experienced higher rates of grade 3 irAEs [13]. They found a treatment-induced decline in circulating B cells and an increase in CD21low B cells and plasmablasts and these changes in B cells preceded and correlated with both the frequency and timing of irAEs.

We are aware that our study has some limitations. Although we included a large cohort, few patients developed the same irAE subtype so as to be studied as a subgroup. It is well known that autoantibody positivity can differ depending on the organ affected. Interestingly, after ICI, autoantibody positivity is frequent in irAEs involving endocrine function, skin and muscle, but rare in irAEs affecting other organs or systems [40]. Additionally, the heterogenicity in the type of tumors, ICI agents and treatment schedules may have influenced the results and conclusions should be taken with caution until the validation of results in larger cohorts. Another limitation is that we do not know the antigenic target against which the autoantibodies react with the analysis of IIF patterns, even though it is a reliable method for screening ANAs. Further studies designed to identify common autoantigens would be of great interest. On the other hand, it is also important to highlight the main strengths of our study. None of the previous studies considered patients with a background of ANA positivity. This novel analysis approach to changes in ANA patterns and not only ANA positivity allowed us to also consider ANA development in patients with a background of ANA positivity, who should not be underestimated.

In conclusion, our findings suggest that antinuclear autoantibody screening during immune checkpoint-blockade therapies could be useful for monitoring patients to predict severe irAEs. This screening could consequently improve the early detection and management of irAEs and avoid treatment interruption. However, further studies integrating humoral and cellular autoreactivity are needed to elucidate the role of these autoantibodies in the pathogenesis of irAEs.

## 4. Materials and Methods

### 4.1. Patients

One hundred and fifty-two consecutive patients diagnosed with solid tumors treated with anti-PD-(L)1 blockade agents alone or in combination with chemotherapy or other immunotherapy drugs at Hospital de la Santa Creu I Sant Pau (Barcelona, Spain) were prospectively included from September 2018 to March 2020 and monitored by the Department of Medical Oncology and the Department of Immunology. The end of the follow-up was September 2020. Patients with prior autoimmune diseases were excluded from the study. All patients included received at least one dose of ICI. We could not collect the pre-treatment (baseline) samples of 10 patients. After the first dose of ICI, eight patients discontinued ICI treatment due to progression criteria and no additional samples were collected after ICI-initiation. Thus, in line with our goal, we included samples at baseline and after ICI initiation from 134 patients.

Written informed consent was obtained from each patient and ethical approval for the study was granted by the Institutional Ethics Committee (Institutional Review Board number: IIBSP-PDL-2017-82). Patients’ data were collected from electronic medical records.

IrAEs were defined as adverse events with a potential immunologic basis that required close monitoring and/or potential intervention with immunosuppressive or hormone replacement. Patient symptoms, physical examination and laboratory data were assessed every 3–4 weeks. Thyroid function was evaluated at baseline and every six weeks thereafter. IrAE severity was graded according to the Common Terminology Criteria for Adverse Events (CTCAE) version 5.0 [41].

### 4.2. Sample Collection

Serial serum samples from each patient were collected at baseline and after initiation of ICI treatment for a maximum of six months of follow-up (at 0, 4, 10, 18 and 24 weeks after ICI initiation). Additionally, when a patient experienced a grade ≥2 irAE, an extra sample was collected. All samples were frozen and stored at −20 °C until autoantibody determination.

### 4.3. Detection of Antinuclear Antibodies

The detection of ANA was based on indirect immune-fluorescence (IIF) in agreement with international recommendations [42]. Assays were performed on a fully automated system (QUANTA-Lyser, Werfen, Spain). Briefly, a patient’s serum was incubated with human epithelial cell line 2 (HEp-2) as a substrate attached to a slide (Innova, Werfen, Spain) to allow the specific binding of autoantibodies to substrate antigens on the slide. After washing, anti-human antibody (IgG) conjugated to fluorescein was added as a detection reagent. Next, the slide was examined under a microscope by trained personnel for fluorescence pattern interpretation according to the international consensus on antinuclear antibody pattern ICAP [42]. Samples with ANA titers equal to or superior to 1:160 were considered positive. All serum samples from a single patient were analyzed with the same batches of reagents to avoid bias in the fluorescence pattern interpretation.

### 4.4. Statistical Analysis

To describe our population, numbers and percentages were used for qualitative variables, while means (± standard error of the mean, SEM) and medians (interquartile ranges, IQR) were calculated for quantitative variables with normal and non-normal distributions, respectively. The Kolmogorov–Smirnov test was applied to analyze the data distribution. Comparisons between groups were tested with Student’s t or the Mann–Whitney test according to normal distribution. ANOVA and Kruskal–Wallis tests were used to compare more than two groups. Fisher and Chi-square tests were used for the comparison of frequencies. The long-rank Mantel–Cox test was used to analyze differences in overall survival (OS), progression-free survival (PFS) and the moment of irAE onset during the follow-up period. All *p* values lower than 0.05 were considered statistically significant. All analyses were performed using Graph Pad Prism 7 software.

## Figures and Tables

**Table 1:** Patient characteristics and ICI treatment comparison in patients grouped according to the presence of ANA during therapy.

	ANA De Novo (*n* = 22)	ANA (*n* = 37)	Non-ANA (*n* = 75)	*p*
**Sex male, n (%)**	17 (77.3)	22 (59.4)	56 (74.7)	0.19
**Age, median (IQR)**	65 (60–76)	71 (63–74.5)	67 (58–77)	0.62
**Tumor type, n (%)**				
NSCLC	17 (77.3)	27 (72.9)	47 (62.7)	0.32
Melanoma	2 (9.1)	4 (10.8)	14 (18.7)	0.38
Renal	1 (4.5)	4 (10.8)	5 (6.7)	0.62
Head and Neck	2 (9.1)	0 (0)	7 (9.3)	0.15
Urothelial	0 (0)	2 (5.4)	2 (2.7)	0.48
**ICI schedule, n (%)**				
*Anti PD-(L)1 in monotheraphy*	17 (77.3)	29 (78.4)	58 (77.3)	0.99
Nivolumab	4 (18.2)	4 (10.8)	17 (22.7)	0.31
Pembrolizumab	9 (40.9)	10 (27)	18 (24)	0.29
Atezolizumab	1 (4.5)	11 (29.7)	12 (16)	0.04
Durvalumab	3 (13.6)	4 (10.8)	6 (8)	0.70
Avelumab	0 (0)	0 (0)	1 (1.3)	0.67
Retifanlimab	0 (0)	0 (0)	4 (5.3)	0.19
*Anti PD-(L)1 in combination with immunotherapy*	4 (18.2)	5 (13.5)	10 (13.3)	0.84
Anti-CTLA4 (Ipilimumab, Tremelimumab)	0 (0)	3 (8.1)	5 (6.7)	0.41
Anti-LAG3 (Eftilagimod)	3 (13.6)	1 (2.7)	4 (5.3)	0.21
Anti-NKG2A (Monalizumab)	0 (0)	1 (2.7)	1 (1.3)	0.69
Anti-CD73 (Oclelumab)	1 (4.5)	0 (0)	0 (0)	0.07
*Anti PD-(L)1 in combination with chemotherapy*	1 (4.5)	3 (8.1)	7 (9.3)	0.77
**Line of treatment, n (%)**				
1st line	11 (50)	17 (45.9)	36 (48)	0.95
≥2nd line	7 (31.8)	17 (45.9)	33 (44)	0.52
Adjuvant	1 (4.5)	1 (2.7)	3 (4)	0.92
Maintenance	3 (13.6)	2 (5.4)	3 (4)	0.24
PD-L1 expression *, n (%)				
Negative (0–1%)	2 (9.1)	2 (5.4)	8 (10.7)	0.65
Low (1–49%)	2 (9.1)	9 (24.3)	12 (16)	0.29
High (≥50%)	10 (45.5)	9 (24.3)	10 (13.3)	0.005

* Tumoral PD-L1 expression was available only in 64 patients.

**Table 2:** Clinical features and ANA patterns in pre- and post-treatment samples of patients who developed ANA de novo during ICI treatment.

Patient	Sex	Age	Tumor	Treatment	Line of Treatment	Pre-Treatment ANA Patterns ^a^ (Titer)	Post-Treatment ANA Patterns ^a^ (Titer)	ANA Development (Days)	irAEs	Grade of Severity ^b^	irAEs Onset ^b^ (Days)
1	M	60	NSCLC	Pembrolizumab + Chemotherapy	Adjuvant	Negative	Cytoplasmic speckled (1:320)	6	Rash, pruritus, hepatotoxicity	1, 1, 2	230, 230, 6
2	M	83	NSCLC	Pembrolizumab	1st	Negative	Nuclear speckled (1:160), Cytoplasmic fibrillar (1:160)	41	Hepatotoxicity	3	41
3	M	58	NSCLC	Pembrolizumab	1st	Negative	Nuclear speckled (1:320), Cytoplasmic fibrillar (1:160), Intercellular Bridge (1:160)	39	Hypertiroidism	1	60
4	F	63	Melanoma	Pembrolizumab	1st	Negative	Nuclear speckled (1:320)	41	Diabetes Mellitus	1	41
5	M	67	NSCLC	Pembrolizumab	2nd	Negative	Nuclear speckled (1:640)	18	Hypophisitis, arthitis	3, 2	259, 207
6	F	66	NSCLC	Durvalumab	Maintenance	Negative	Cytoplasmic fibrillar (1:640), NuMa-like (1:640)	113	No	_	_
7	F	73	NSCLC	Pembrolizumab + Eftilagimod	1st	Negative	Cytoplasmic dots (1:160)	41	No	_	_
8	M	61	NSCLC	Durvalumab	Maintenance	Negative	Nuclear speckled (1:160), Spindle fibers (1:160)	35	Pruritus, mucositis	1,1	91, 11
9	M	62	Neck and Head Carcinoma	Nivolumab	3rd	Negative	Nuclear speckled (1:160)	53	No	_	_
10	M	64	NSCLC	Pembrolizumab	1st	Negative	Nuclear speckled (1:160)	70	No	_	_
11	M	76	NSCLC	Durvalumab + Oleclumab	Maintenance	Negative	Cytoplasmic fibrillar (1:1280)	27	Pneumonitis	3	55
12	M	39	NSCLC	Durvalumab	2nd	Negative	Cytoplasmic fibrillar (1:1280)	53	Pruritus	1	9
13	F	64	NSCLC	Atezolizumab	3rd	Negative	Nuclear speckled (1:160)	41	Colitis	3	108
14	M	76	Renal Cancer	Nivolumab	2nd	Negative	Cytoplasmic speckled (1:160)	111	No	_	_
15	M	76	NSCLC	Pembrolizumab	2nd	Cytoplasmic speckled (1:320)	Cytoplasmic speckled (1:160), Rods & Rings (1:1280)	49	Pneumonitis, hepatotoxicity	4,4	196, 196
16	M	60	NSCLC	Pembrolizumab	1st	Nuclear speckled (1:160)	Nuclear speckled (1:160), Nucleolar (1:160)	126	No	_	_
17	F	84	Melanoma	Nivolumab	1st	Nuclear speckled (1:160)	Nuclear speckled (1:320), Cytoplasmic speckled (1:320)	68	Pruritus, hypothiroidism	1,2	40, 83
18	M	78	NSCLC	Pembrolizumab	1st	Nuclear speckled (1:160)	Nuclear speckled (1:160), Nucleolar (1:160)	121	No	_	_
19	M	60	NSCLC	Nivolumab	1st	Cytoplasmic reticular (1:640)	Cytoplasmic reticular (1:160), Nuclear speckled (1:320)	118	Hepatotoxicity	3	167
20	M	59	Neck and Head Carcinoma	Pembrolizumab	2nd	PCNA-like (1:640), Nucleolar (1:640)	PCNA-like (1:640), Nucleolar (1:640), Intercellular bridge (1:320)	167	No	_	_
21	M	73	NSCLC	Pembrolizumab + Eftilagimod	1st	Nuclear coarse speckled (1:640)	Nuclear fine dense speckled (1:320)	43	Rash, pruritus, colitis	2, 1, 1	7, 7, 203
22	M	67	NSCLC	Pembrolizumab + Eftilagimod	1st	Nuclear speckled (1:320), Centromeric CENP-F-like (1:160)	Nuclear speckled (1:320), Centromeric CENP-F-like (1:160), Nucleolar (1:320)	105	Rash, pruritus, psoriasis	2, 2, 2	33, 105, 11

a. Nomenclature of ANA patterns according to a competent level of ICAP includes: Nuclear speckled (AC-4,5); Nucleolar (AC-8, 9, 10); CENP-F-like (AC-14); Cytoplasmic fibrillar (AC-15, 16, 17); Cytoplasmic speckled (AC-19, 20); Rods&Rings (AC-23); Spindle fibers (AC-25); NuMa-like (AC-26); Intercellular bridge (AC-27). b. Data of grade and day of onset are in the same order as their related irAE type.

## Data Availability

The experimental data generated during the current study are available from the corresponding author on reasonable request.

## Supplementary Materials

The following supporting information can be downloaded at: https://www.mdpi.com/article/10.3390/ijms232012641/s1.
