# Supplementary material for: Association between Changes in the Patterns of Antinuclear Autoantibodies during Immune Checkpoint Inhibition Therapy and the Development of Severe Immune Related Adverse Events
Source: Int J Mol Sci. 2022 Oct 20;23(20):12641. doi: 10.3390/ijms232012641 (PMC9604501; doi:10.3390/ijms232012641)
Supplement: Supplementary file 1 [file ijms-23-12641-s001.zip › ijms-1972103-supplementary.pdf]

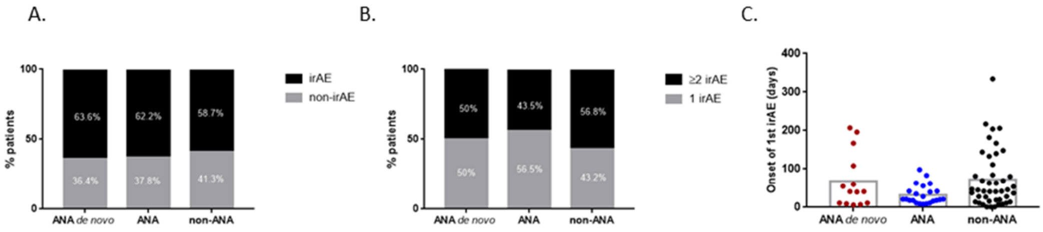

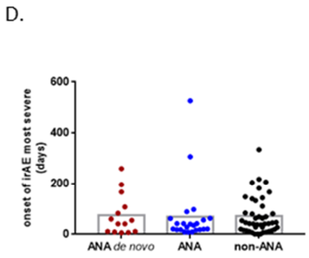

E.

Type of irAE	ANA *de novo* (*n* = 22)	ANA (*n* = 37)	Non-ANA (*n* = 75)	*p*
Pruritus, *n* (%)	6 (27.2)	14 (37.8)	28 (37.3)	0.65
Rash, *n* (%)	3 (13.6)	8 (21.6)	10 (13.3)	0.5
Endocrine, *n* (%)	4 (18.2)	7 (18.9)	10 (13.3)	0.7
Colitis, *n* (%)	2 (9.1)	3 (8.1)	5 (6.7)	0.91
Arthralgias, *n* (%)	1 (4.5)	2 (5.4)	6 (8)	0.79
Hepatitis, *n* (%)	4 (18.2)	5 (13.5)	3 (4)	0.06
Pneumonitis, *n* (%)	2 (9.1)	1 (2.7)	6 (8)	0.51
Mucositis, *n* (%)	1 (4.5)	4 (10.8)	3 (4)	0.34

Figure S1. Comparison of irAE manifestation in patients grouped according to the presence of ANA. Association of ANA presence with (**A)** irAE development, (**B**) number of irAEs developed by each patient, (**C**) time of onset of the first and (**D**) most severe irAE developed, and (**E**) type of irAE. Chi-square test was used for statistical analysis of frequencies. Mann-Whitney test was used for comparing times of irAE onset.

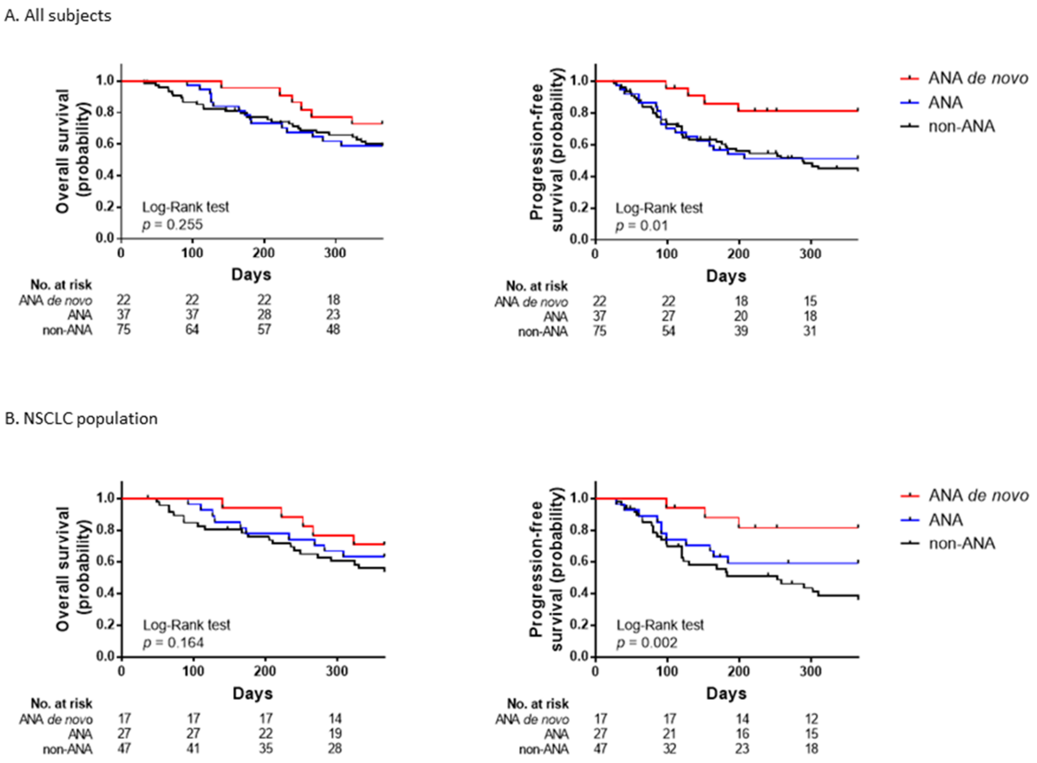

Figure S2: Comparison of treatment response in patients grouped according to the presence of ANA. Overall survival and progression-free survival analysis in (**A**) all patients included in the study and (**B**) NSCLC patients. Log-rang Mantel-Cox test was used for analysis of patients groups during 365 days of follow-up.
